# Supplementary material for: Using information of relatives in genomic prediction to apply effective stratified medicine
Source: Sci Rep. 2017 Feb 9;7:42091. doi: 10.1038/srep42091 (PMC5299615; doi:10.1038/srep42091)
Supplement: Supplementary Information [file srep42091-s1.pdf]

Supplementary data

# Using information of relatives in genomic prediction to apply effective stratified medicine

S. Hong Lee, W.M. Shalanee P. Weerasinghe, Naomi R. Wray, Michael E. Goddard, and Julius H.J. van der Werf

Supplementary Note

**Odds ratio in percentile analysis derived from truncated normal theory**
For a disease or disorder, it is assumed that there are normalised individual risk profile scores ($u$) for the sample that can be estimated from GBLUP[1; 2]. When selecting the top X proportion of the risk profile scores, the expectation and variance are[3; 4]

$$E(u \mid top) = i_{top} \cdot R$$
$$Var(u \mid top) = \sigma_{top}^2 = [1 - i_{top} \cdot (i_{top} - t_{top})] \cdot R^2$$

where $i_{top}$ is the mean risk scores for the top selected group according to the risk profile score, $R^2$ is the proportion of the total variance on the liability scale explained by the risk profile scores and $t_{top}$ is the threshold on the normal distribution which truncates the proportion of the top risk group. Considering the vector of $u$, the probability density (z) is the height of the normal curve at the threshold, $t_{top}$. If we define X = the proportion of the top risk group according to the risk profile score, $i_{top}$ = z / X. According to truncated normal distribution theory[3; 4], the probability of being a case for the top risk group is

$$P(case \mid top) \approx \left[1 - \Phi\left[(t_{top} - i_{top} \cdot R) / \sqrt{\sigma_{top}^2 + (1 - R^2)}\right]\right] .$$

Similarly, the probability being a case for the bottom risk group is

$$P(case \mid bottom) \approx \left[1 - \Phi\left[(t_{bottom} - i_{bottom} \cdot R) / \sqrt{\sigma_{top}^2 + (1 - R^2)}\right]\right]$$

where $i_{bottom}$ is the mean risk scores for the bottom selected group and $t_{bottom}$ is the threshold on the normal distribution which truncates the proportion of the risk profile scores of the bottom risk group. Therefore, the odds ratio of expected case-control status by contrasting the top and bottom percentile (equation (4)) and the top and the normal population (equation (5)) can be obtained.

**Effective number of chromosome segments with a genomic length of *L* Morgan**
With an $L$ Morgan region, the sum in equation (7) can be written as

$$\sum_{i=1}^{M}\sum_{j=1}^{M} r_{i,j}^2 \Big/ M^2 = \sum_{i=0}^{M-1} 1/[1 + 4N_e L(i/(M-1))] \cdot 2(1 - i/M) \cdot (1/M)$$

This can be transformed to a function of x with infinity data points ranging from 0 to 1 as

$$f(x) = 1/[1 + 4N_e L \cdot x] \cdot 2(1 - x) .$$

Integrating this function over $x$ ranging from 0 to 1 gives the mean of the function, i.e. $\sum_{i=1}^{M}\sum_{j=1}^{M} r_{i,j}^2 \Big/ M^2$ can be obtained as

$$\int_0^1 f(x)dx = [\ln(4N_eL+1) + 4N_eL(\ln(4N_eL+1)-1)]/(8N_e^2L^2).$$

### $M_e$ from the genomic relationship matrix

$M_e$ can also be estimated from a genomic relationship matrix (GRM). In this derivation, the elements in the GRM are $A_{ij} = \sum_{m=1}^{M} x_{im}x_{jm} / M$ where $x_{im}$ and $x_{jm}$ are the standardised genotype coefficients (mean 0 and variance 1) for the $i$th and $j$th individuals at the $m$th locus. It is possible to construct a GRM for each locus, and the elements in the GRM at the $m$th locus are $A_{ij(m)} = x_{im}x_{jm}$. Then, the variance of the mean of $A_{ij(m)}$ across all the SNPs is

$$\text{var}\left(\sum_{m=1}^{M} A_{ij(m)} / M\right) = \frac{1}{M^2}\left[\sum_{m=1}^{M}\sum_{l=1}^{M} \text{cov}(A_{ij(l)}, A_{ij(m)})\right]. \quad (13)$$

When two loci are correlated such that the correlation between the $m$th and $l$th locus is $cor(x_{im}, x_{il}) = r_{ml}$, the correlation between $A_{ij(m)}$ and $A_{ij(l)}$ is $cor(A_{ij(m)}, A_{ij(l)}) \cong r_{ml}^2$ when individuals $i$ and $j$ are randomly sampled from the population ($i \neq j$). The derivation is in the following – the expectation of the relationship between the $i$th and $j$th individual at $m$th locus is

$$\begin{aligned}
E(A_{ij(m)}) &= E(x_{im}x_{jm}) \\
&= \frac{x_{1m}(x_{1'm} + ... + x_{N'm}) + ... + x_{Nm}(x_{1'm} + ... + x_{N'm})}{NN'} \\
&= \frac{(x_{1m} + ... + x_{Nm})(x_{1'm} + ... + x_{N'm})}{NN'} \\
&= E(x_{im})E(x_{jm}) \\
&= 0
\end{aligned}$$

while the variance of the relationship between the $i$th and $j$th individual at the $m$th locus is

$$\begin{aligned}
\text{var}(A_{ij(m)}) &= \text{var}(x_{im}x_{jm}) \\
&= \frac{x_{1m}^2(x_{1'm}^2 + ... + x_{N'm}^2) + ... + x_{Nm}^2(x_{1'm}^2 + ... + x_{N'm}^2)}{NN'} \\
&= \frac{(x_{1m}^2 + ... + x_{Nm}^2)\ (x_{1'm}^2 + ... + x_{N'm}^2)}{NN'} \\
&= \text{var}(x_{im})\text{var}(x_{jm}) \\
&= 1
\end{aligned}$$

and, the covariance of the pairwise relationship between the $m$th and $l$th loci is

$$\begin{aligned}\text{cov}(A_{ij(m)}, A_{ij(l)}) &= \text{cov}(x_{im}x_{jm}, x_{il}x_{jl}) \\ &= \frac{x_{1m}x_{1l}(x_{1'm}x_{1'l} + \ldots + x_{N'm}x_{N'l}) + \ldots + x_{Nm}x_{Nl}(x_{1'm}x_{1'l} + \ldots + x_{N'm}x_{N'l})}{NN'} \\ &= \frac{(x_{1m}x_{1l} + \ldots + x_{Nm}x_{Nl})(x_{1'm}x_{1'l} + \ldots + x_{N'm}x_{N'l})}{NN'} \\ &= \text{cov}(x_{im}, x_{il})\text{cov}(x_{jm}, x_{jl}) \\ &= r_{ml}^2\end{aligned}$$

.

Therefore, replacing $\text{cov}(A_{ij(m)}, A_{ij(l)})$ with $r_{ml}^2$ , equation (13) is equivalent to the denominator in equation (7). Hence, $M_e$ can be approximated as

$$M_e = 1 \Big/ \text{var}\left(\sum_{m=1}^{M} A_{ij(m)} / M\right).$$

This was also reported by Goddard et al. (2011)[5] showing that the variance of the genomic relationships based on genome-wide SNPs is the mean of the $r^2$ measure of LD.

This same procedure can be applied to subsets of SNPs, e.g. SNPs grouped according to the chromosomes, and can be rewritten as

$$M_e = 1 \Big/ \text{var}\left(\sum_{k=1}^{N_{chr}} A_{ij(k)} / M\right)$$

where $N_{chr}$ is the number of chromosomes and $A_{ij(k)}$ is the GRM estimated based on the SNPs located on the $k$th chromosome.

Supplementary Table 1. Comparison between observed $M_e$ (from equation (6) and (7)) and expected $M_e$ (from equation (8)) using the coalescence function, $r^2 = 1 / (1 + 4N_e \times c)$.

| Parameters | equation 6 | equation 7 | equation 8 |
|---|---|---|---|
| When varying the effective population size, L=1 and M=10000 | | | |
| Ne=50 | 23.57 | 23.1 | 23.1 |
| Ne=100 | 40.52 | 39.93 | 39.93 |
| Ne=200 | 71.02 | 70.24 | 70.25 |
| Ne=500 | 152.47 | 151.32 | 151.4 |
| Ne=1000 | 275.21 | 273.62 | 274.11 |
| When varying the genomic length, Ne=500 and M=10000 | | | |
| L=0.3 | 55.46 | 55.43 | 56.11 |
| L=0.5 | 85.32 | 84.47 | 84.52 |
| L=1 | 152.47 | 151.32 | 151.4 |
| L=1.5 | 215.3 | 214 | 213.9 |
| L=2 | 275.6 | 273.98 | 274.11 |
| When varying the number of SNPs, Ne=500 and L=1 | | | |
| M=500 | 134.94 | 134.29 | 151.4 |
| M=1000 | 146.36 | 145.44 | 151.4 |
| M=2000 | 150.71 | 149.65 | 151.4 |
| M=5000 | 152.22 | 151.1 | 151.4 |
| M=10000 | 152.47 | 151.32 | 151.4 |

Equation (6), (7) and (8) were confirmed with actual analyses (i.e. inverse and summation) of a squared correlation matrix among SNPs. The effective population size $N_e$ = 50, 100, 200, 500 or 1000, the genomic length $L$ = 0.3, 0.5, 1, 1.5 or 2 Morgan, and the number of SNPs (equally) spanning the genomic region $M$ = 500, 1000, 2000, 5000 or 10000 were used. A squared correlation matrix was constructed for SNPs using the coalescence function, $r^2 = 1 / (1 + 4N_e \times c)$[6] where $N_e$ is known and $c$ is the distance in Morgan between each pair of SNPs. Using the inverse and summation of the SNP squared correlation matrix, observed $M_e$ was obtained from equation (6) and (7). As well, equation (8) was used to obtain the expected $M_e$ given $N_e$ and $L$. The observed and expected $M_e$ values were agreed very well with various values for $N_e$ and $M_e$. For a smaller $M$ (< 1000), the observed and expected $M_e$ became different, which was expected because equation (8) was derived based on the assumption of the number of SNPs ($M$), being infinity.

Supplementary Table 2. Comparison between observed $M_e$ (from equation (6) and (7)) and expected $M_e$ (from equation (9)) using the alternative coalescence function, $r^2 = 1 / (2 + 4N_e \times c)$.

| Parameters | equation 6 | equation 7 | equation 9 |
|---|---|---|---|
| When varying the effective population size, L=1 and M=10000 | | | |
| Ne=50 | 28.07 | 27.31 | 27.31 |
| Ne=100 | 47.13 | 46.19 | 46.19 |
| Ne=200 | 81.05 | 79.85 | 79.86 |
| Ne=500 | 170.72 | 169.02 | 169.04 |
| Ne=1000 | 304.94 | 302.64 | 302.79 |
| When varying the genomic length, Ne=500 and M=10000 | | | |
| L=0.3 | 64.53 | 63.47 | 63.48 |
| L=0.5 | 96.89 | 95.6 | 95.62 |
| L=1 | 170.72 | 169.02 | 169.04 |
| L=1.5 | 239.39 | 237.36 | 237.39 |
| L=2 | 305.07 | 302.75 | 302.79 |
| When varying the number of SNPs, Ne=500 and L=1 | | | |
| M=500 | 162.93 | 161.65 | 169.04 |
| M=1000 | 168.38 | 166.86 | 169.04 |
| M=2000 | 170.09 | 168.46 | 169.04 |
| M=5000 | 170.63 | 168.94 | 169.04 |
| M=10000 | 170.72 | 169.02 | 169.04 |

Equations (6), (7) and (9) were confirmed with actual analyses (i.e. inverse and summation) of the squared correlation matrix among SNPs. The effective population size $N_e$ = 50, 100, 200, 500 or 1000, the genomic length $L$ = 0.3, 0.5, 1, 1.5 or 2 Morgan, and the number of SNPs (equally) spanning the genomic region $M$ = 500, 1000, 2000, 5000 or 10000 were used. A squared correlation matrix was constructed for SNPs using the coalescence function, $r^2 = 1 / (2 + 4N_e \times c)$[7] where $N_e$ is known and $c$ is the distance in Morgan between each pair of SNPs. Using the inverse and summation of the SNP squared correlation matrix, observed $M_e$ was obtained from equations (6) and (7). As well, equation (9) were used to obtain the expected $M_e$ given $N_e$ and $L$. The observed and expected $M_e$ values were agreed very well with various values for $N_e$ and $M_e$. For a smaller $M$ (< 1000), the observed and expected $M_e$ became different, which was expected because equation (9) was derived based on the assumption of the number of SNPs ($M$), being infinity.

Supplementary Table 3. Comparison between expected $M_e$ from (equation (10) and (11)) in this study and those from previous studies.

| Ne | equation 10 1/(1+4Ne*c) | equation 11 1/(2+4Ne*c) | Goddard 2009[a] | Goddard et al. 2011[b] | Meuwissen et al. 2013[c] |
|---|---|---|---|---|---|
| | $N_{chr}$=1 and $L$ = 1 (genomic length of 1 Morgan) | | | | |
| 50 | 23 | 27 | 19 | 26 | 22 |
| 100 | 40 | 46 | 33 | 43 | 38 |
| 200 | 70 | 80 | 60 | 75 | 67 |
| 500 | 151 | 169 | 132 | 161 | 145 |
| 1000 | 274 | 303 | 241 | 290 | 263 |
| | $N_{chr}$=5 and $L$ = 1 (genomic length of 5 Morgan) | | | | |
| 50 | 71 | 79 | 94 | 128 | 109 |
| 100 | 130 | 143 | 167 | 217 | 189 |
| 200 | 239 | 261 | 299 | 377 | 334 |
| 500 | 539 | 583 | 658 | 805 | 724 |
| 1000 | 1004 | 1079 | 1206 | 1448 | 1316 |
| | $N_{chr}$=30 and $L$ = 1 (genomic length of 30 Morgan) | | | | |
| 50 | 127 | 130 | 566 | 767 | 651 |
| 100 | 246 | 254 | 1001 | 1303 | 1132 |
| 200 | 479 | 493 | 1795 | 2265 | 2003 |
| 500 | 1157 | 1188 | 3947 | 4827 | 4343 |
| 1000 | 2253 | 2313 | 7234 | 8686 | 7894 |

[a]In the derivation, $r^2 = 1 / (1 + 4N_e \times c)$ was used. [b]$r^2 = 1 / (2 + 4N_e \times c)$ was used. [c]Not mentioned which function was used.

In previous studies[5; 8; 9], there is an inconsistency. In 2009[8], the derived formula was $M_e = 2N_eLN_{chr} / \ln(4N_eL)$ that was changed to $M_e = 2N_eLN_{chr} / \ln(N_eL)$ in 2011[5] and subsequently altered to $M_e = 2N_eLN_{chr} / \ln(2N_e)$ in 2013[9]. In supplementary Table 3 above, there are differences among the values from the previous studies[5; 8; 9]. Compared to the values from equation (10) or (11) in this study, there is non-negligible difference especially when using multiple chromosomes (Supplementary Table 3). Equation (10) and (11) in this study have been verified by an analytical approach (Supplementary Tables 1 and 2) and stochastic simulations (Supplementary Figures 1-3).

Supplementary Table 4. Expected prediction performance for case-control data when the number of records or true heritability varies. The effective population size was $N_e$=100, the length of the genome was 30 Morgan (30 chromosomes each with 1 Morgan long), the population prevalence was $K$=0.1 and the proportion of cases in the sample was $P$=0.5.

| | AUC | OR contrasting the top and bottom 20% | OR contrasting the top 1% and normal population |
|---|---|---|---|
| When varying the sample size in validation set (with $h^2$=0.5) | | | |
| $N$=3000 | 0.85 | 131.99 | 23.01 |
| $N$=6000 | 0.86 | 190.88 | 27.51 |
| $N$=12000 | 0.87 | 237.15 | 30.53 |
| $N$=24000 | 0.88 | 267.00 | 32.32 |
| When varying the heritability (with $N$=3000) | | | |
| $h^2$=0.5 | 0.85 | 131.99 | 23.01 |
| $h^2$=0.6 | 0.88 | 396.77 | 39.04 |
| $h^2$=0.7 | 0.91 | 1513.75 | 74.14 |
| $h^2$=0.8 | 0.93 | 8444.51 | 175.57 |

Supplementary Table 5. The expected accuracy or AUC of genomic prediction from a design with smaller or larger $N_e$ values in the Framingham data when varying underlying true heritability.

| | Quantitative traits | | Case-control | | | |
|---|---|---|---|---|---|---|
| | Expected accuracy | | Expected AUC | | Expected OR[a] | |
| $h^2$ | Small $N_e$ ($M_e$=4434) | Large $N_e$ ($M_e$=31080) | Small $N_e$ ($M_e$=3247) | Large $N_e$ ($M_e$=29480) | Small $N_e$ ($M_e$=3247) | Large $N_e$ ($M_e$=29480) |
| 0.1 | 0.084 | 0.033 | 0.524 | 0.508 | 1.27 | 1.08 |
| 0.2 | 0.163 | 0.065 | 0.548 | 0.516 | 1.62 | 1.18 |
| 0.3 | 0.237 | 0.098 | 0.571 | 0.524 | 2.05 | 1.27 |
| 0.4 | 0.306 | 0.129 | 0.594 | 0.532 | 2.60 | 1.38 |
| 0.5 | 0.372 | 0.161 | 0.617 | 0.540 | 3.30 | 1.50 |
| 0.6 | 0.435 | 0.192 | 0.639 | 0.549 | 4.21 | 1.62 |
| 0.7 | 0.494 | 0.223 | 0.660 | 0.557 | 5.39 | 1.76 |
| 0.8 | 0.551 | 0.254 | 0.682 | 0.565 | 6.95 | 1.91 |
| 0.9 | 0.606 | 0.284 | 0.702 | 0.573 | 9.04 | 2.07 |

[a]The odds ratio of case-control status comparing each 20 percentile to the bottom 20% of the ranked genetic profile scores

Supplementary Table 6. Based on the Framingham data, the accuracy of genomic prediction from a design with smaller or larger $N_e$ values when using body mass index phenotypes.

| | Small $N_e$ | Large $N_e$ |
|---|---|---|
| Quantitative traits (height) - 3394 discovery, 849 validation | | |
| $M_e$ | 4434 | 31080 |
| Expected accuracy | 0.330[a] | 0.046[b] |
| Observed accuracy | 0.349 (0.027) | 0.056 (0.0368) |
| | | |
| Case-control (10% selection); 680 discovery, 170 validation (*K*=0.1 and *P*=0.5) | | |
| $M_e$ | 3247 | 29480 |
| Expected AUC | 0.608[a] | 0.511[b] |
| Observed AUC | 0.618 (0.041) | 0.529 (0.033) |

[a]Expected accuracy from equation (2) using the value for $M_e$ and $h^2$=0.46[10; 11] that is from family studies. [b]Expected accuracy from equation (2) using the value for $M_e$ and $h^2$=0.14[12; 13] that is from population studies. SD over 100 cross-validation replicates is in the bracket.

A

B

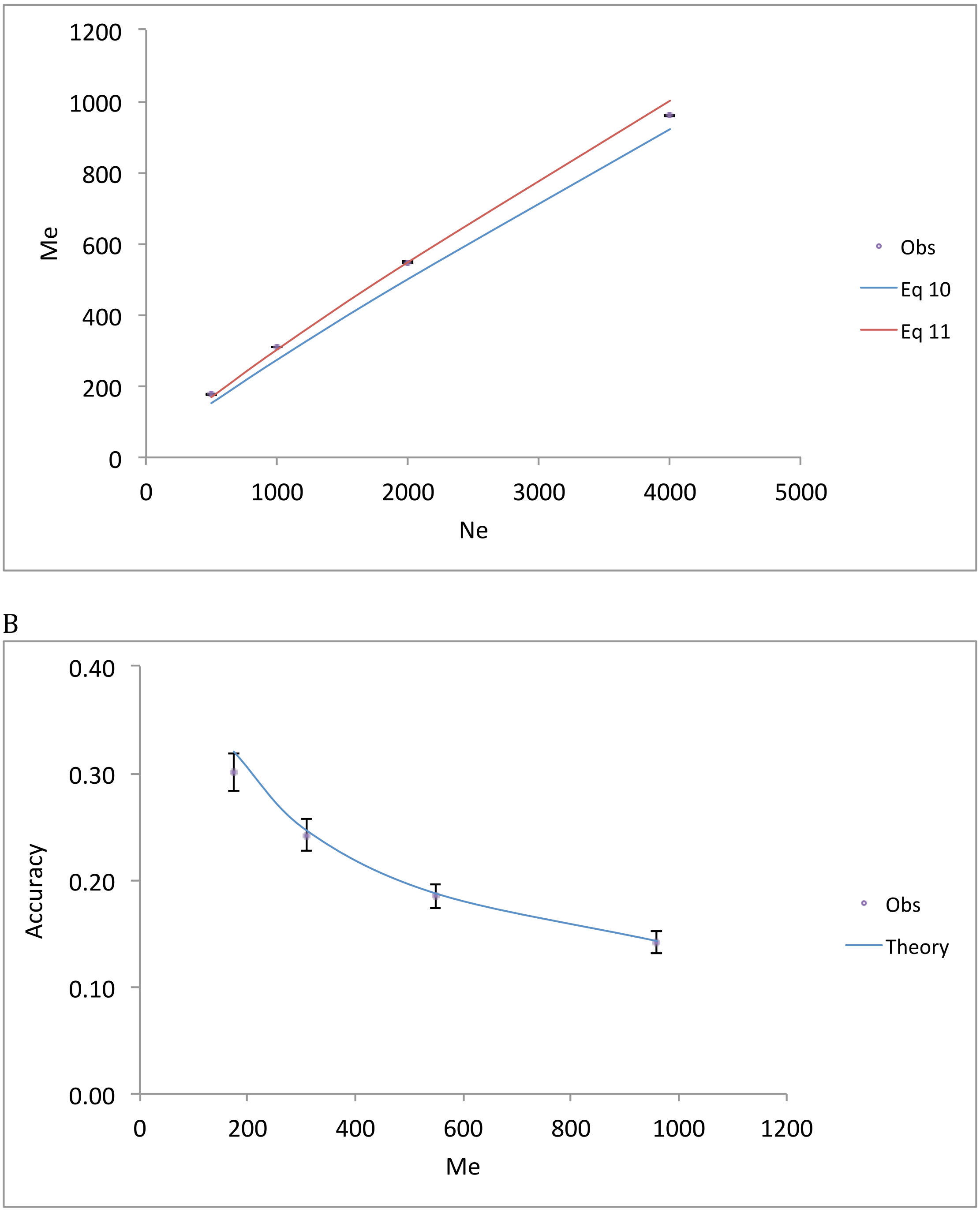


Supplementary Figure 1. Observed Me (Obs) and expected Me from equation (10) and (11) (A) and the confidence interval of observed accuracy (Obs) and expected accuracy from the theory (B) when using a stochastic gene-dropping method across a single chromosome of $L$=1 Morgan with $N_e$ = 500, 1000, 2000 and 4000 for 500, 1000, 2000 and 4000 generations to generate 2000 individuals with genotype and phenotype data in the discovery data set.

A

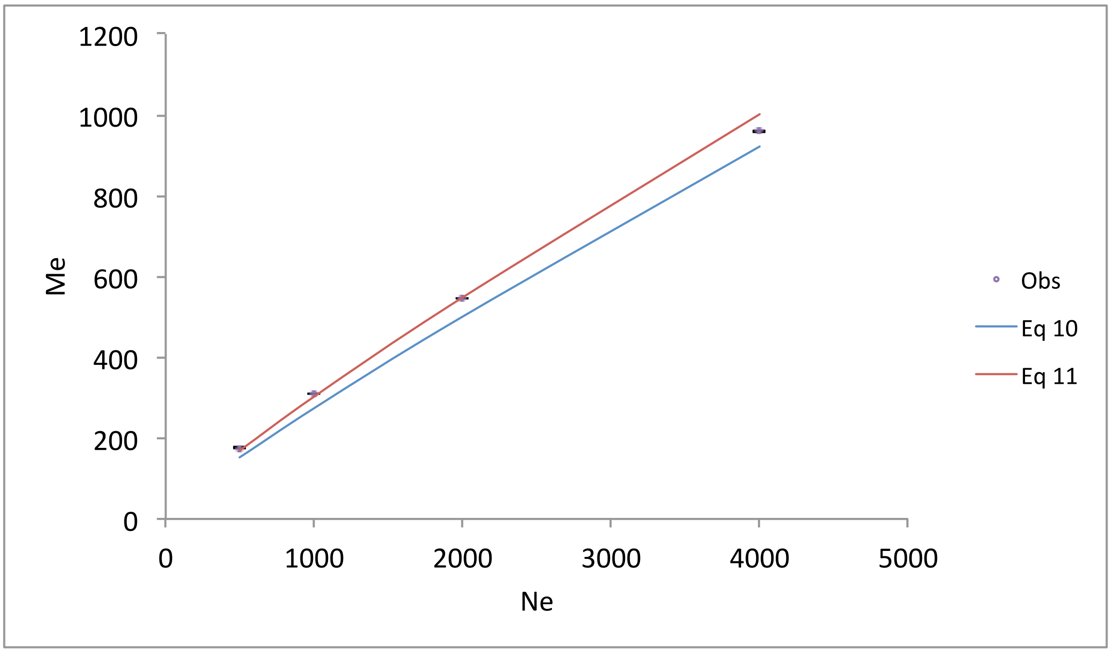


B

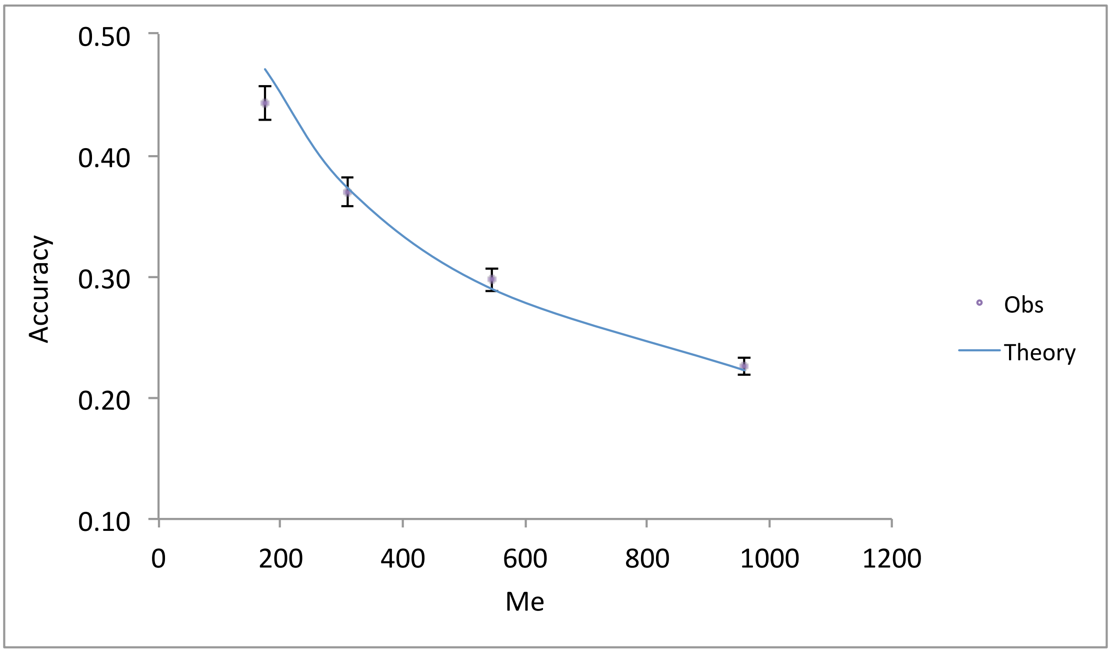


Supplementary Figure 2. Observed Me (Obs) and expected Me from equation (10) and (11) (A) and the confidence interval of observed accuracy (Obs) and expected accuracy from the theory (B) when using a stochastic gene-dropping method across a single chromosome of $L$=1 Morgan with $N_e$ = 500, 1000, 2000 and 4000 for 500, 1000, 2000 and 4000 generations to generate 5000 individuals with genotype and phenotype data in the discovery data set.

A

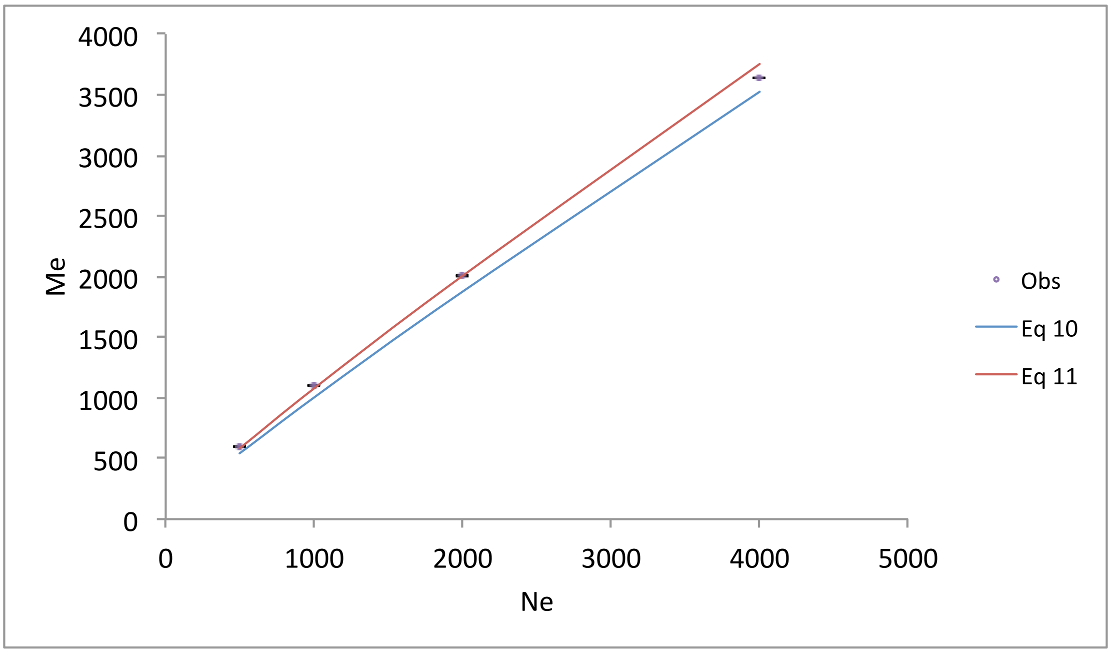


B

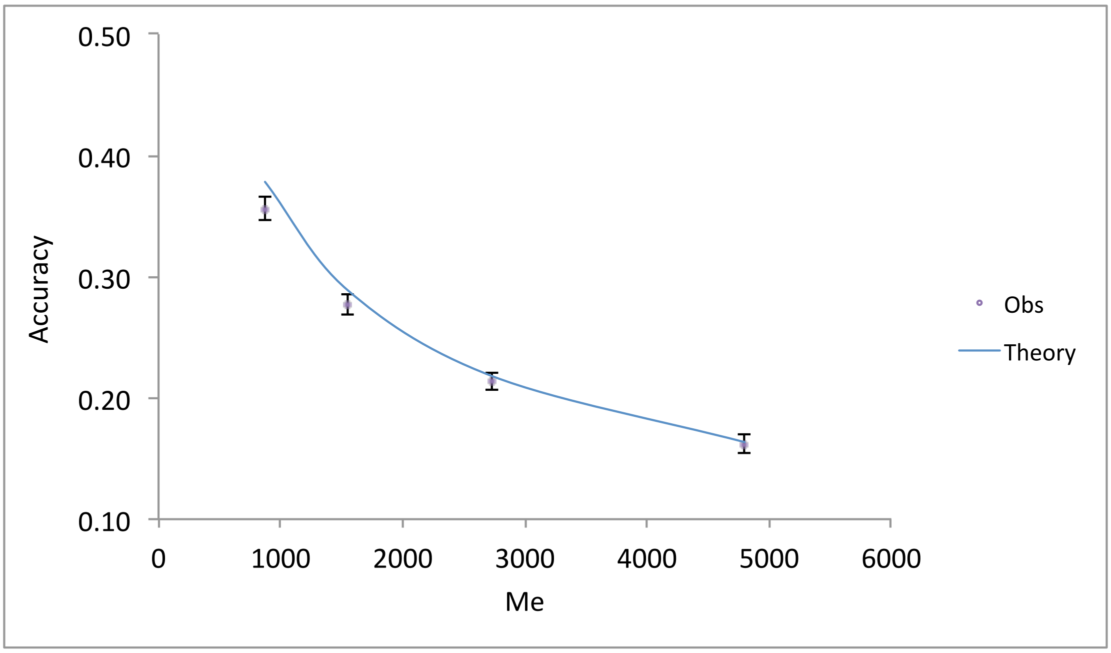


Supplementary Figure 3. Observed Me (Obs) and expected Me from equation (10) and (11) (A) and the confidence interval of observed accuracy (Obs) and expected accuracy from the theory (B) when using a stochastic gene-dropping method across five chromosome, each with $L$=1 Morgan with $N_e$ = 500, 1000, 2000 and 4000 for 500, 1000, 2000 and 4000 generations to generate 2000 individuals with genotype and phenotype data in the discovery data set.

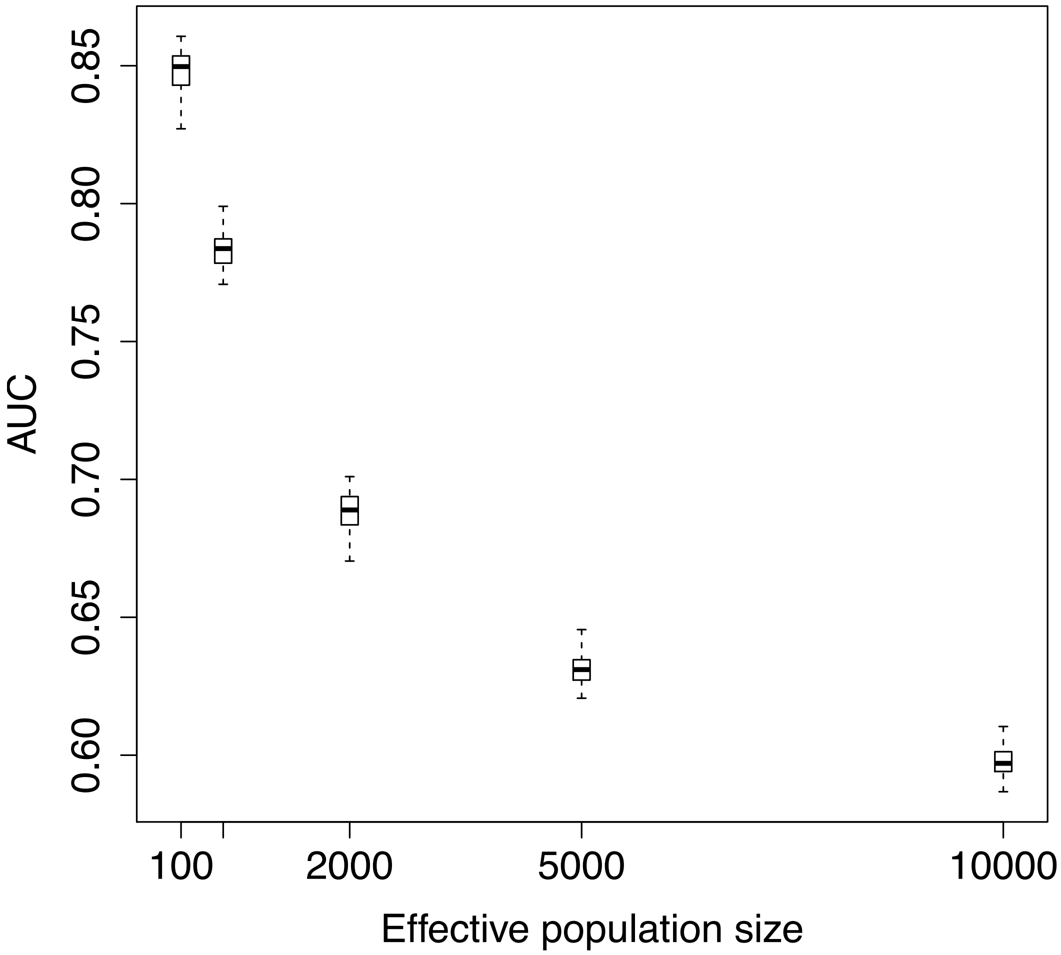


Supplementary Figure 4. Observed AUC from simulated data using the same parameters to obtain the AUC values from the theory (Figure 2). The number of records (*N*) is 3000, the true heritability is 0.5 and a disease or disorder with population lifetime prevalence of *K*=0.1 and a proportion of cases in the sample of *P*=0.5 is used. The observed values are in excellent agreement with the expected values that are 0.85, 0.78, 0.69, 0.63 and 0.60 for the effective population size of 100, 500, 2000, 5000 and 10000.

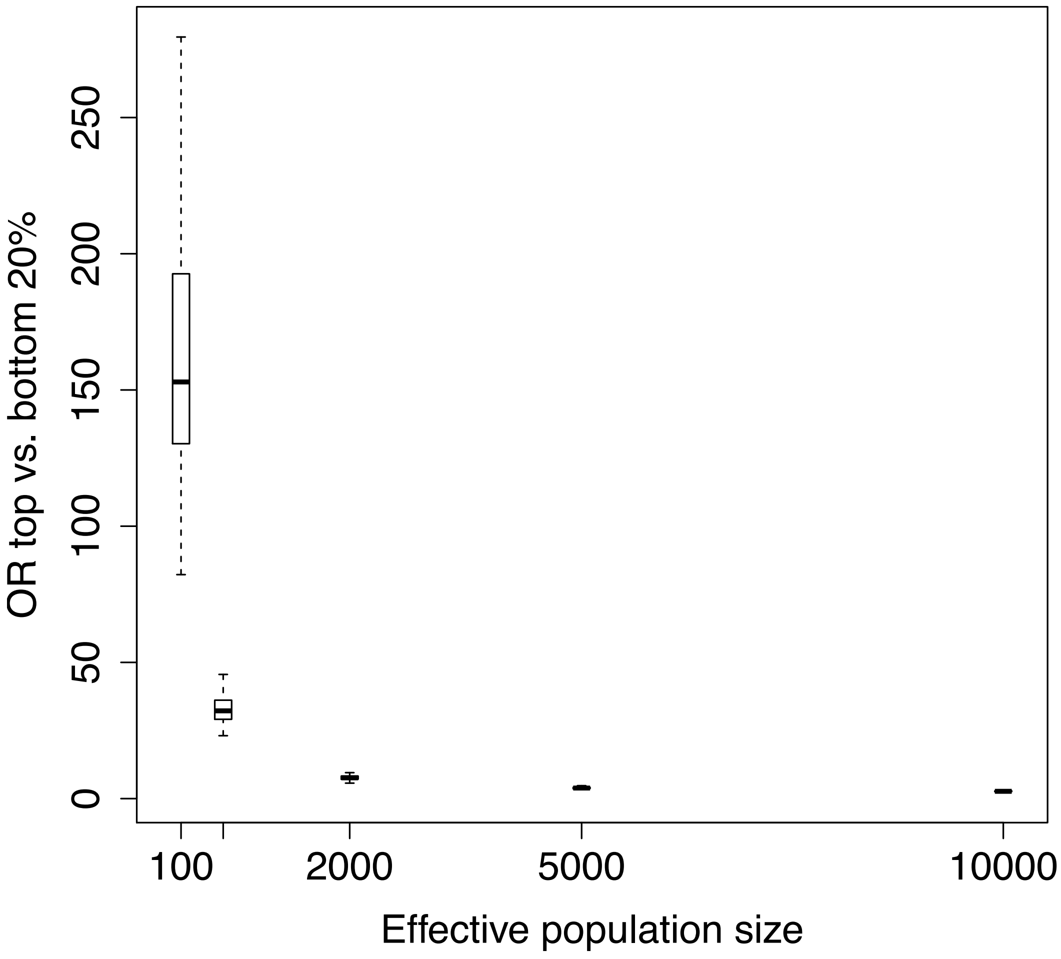


Supplementary Figure 5. Observed odd ratio contrasting the top and bottom 20% of the risk profile scores from simulated data using the same parameters to obtain the odds ratio from the theory (Figure 3). The number of records (*N*) is 3000, the true heritability is 0.5 and a disease or disorder with population lifetime prevalence of *K*=0.1 and a proportion of cases in the sample of *P*=0.5 is used. The observed values are in good agreement with the expected values that are 131.9, 31.0, 7.7, 3.9 and 2.7 for the effective population size of 100, 500, 2000, 5000 and 10000.

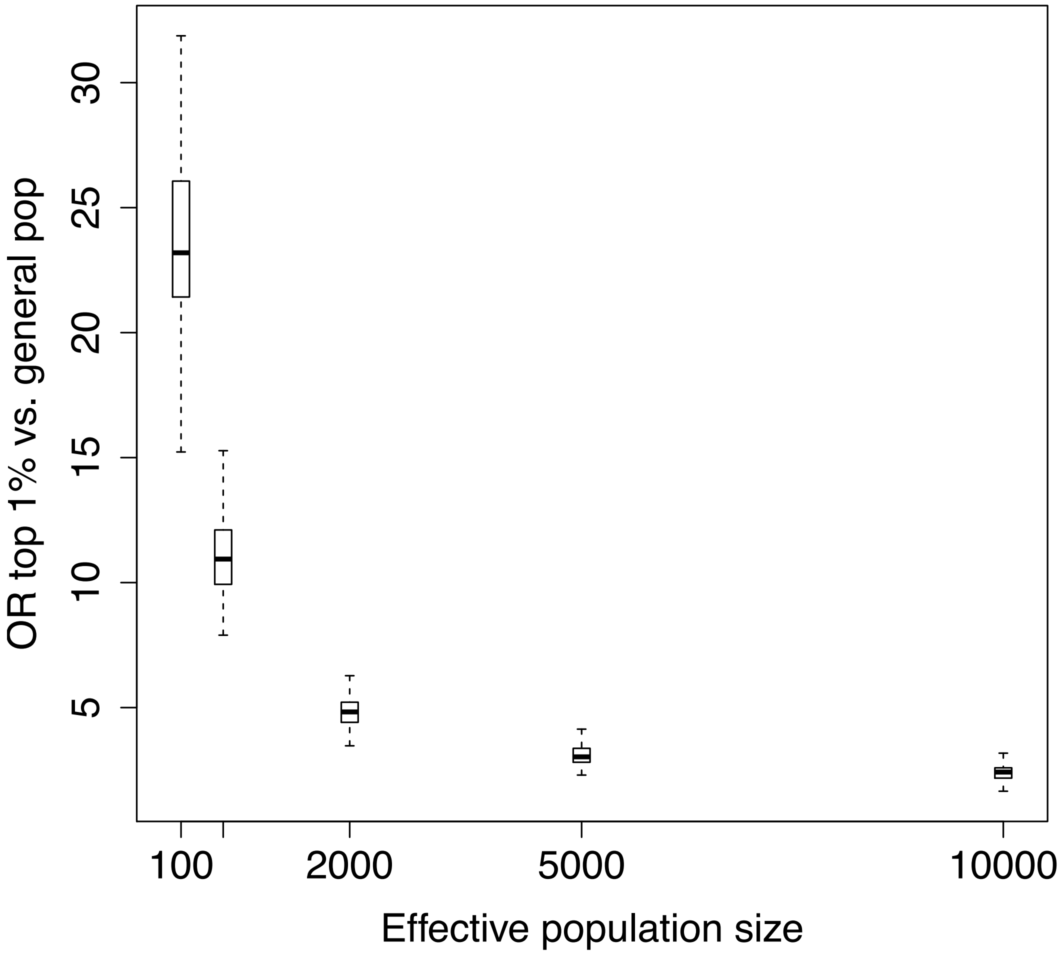


Supplementary Figure 6. Observed odd ratio contrasting the top 1% of the risk profile scores and the general population from simulated data using the same parameters to obtain the odds ratio from the theory (Figure 4). The number of records (*N*) is 3000, the true heritability is 0.5 and a disease or disorder with population lifetime prevalence of *K*=0.1 and a proportion of cases in the sample of *P*=0.5 is used. The observed values are in good agreement with the expected values that are 23.0, 11.0, 5.0, 3.1 and 2.4 for the effective population size of 100, 500, 2000, 5000 and 10000.

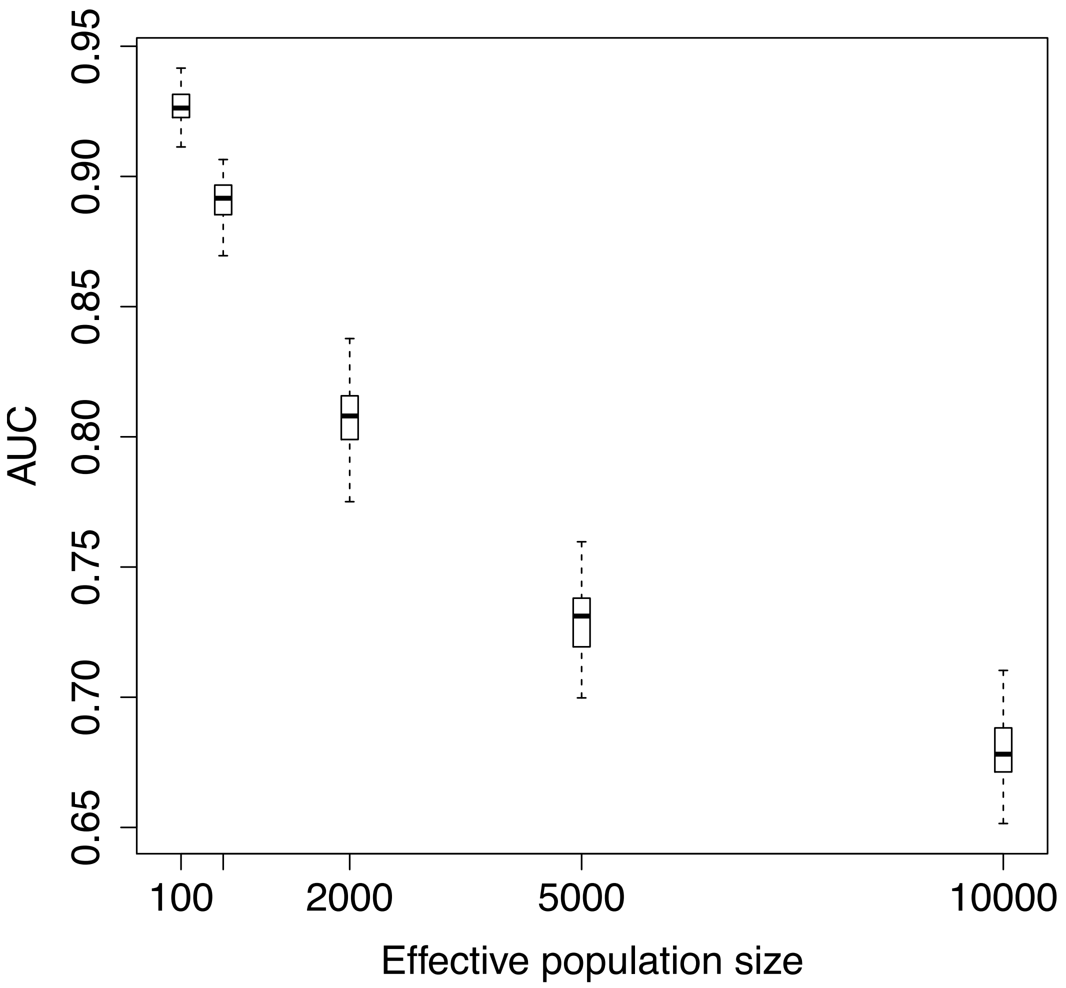


Supplementary Figure 7. Observed AUC from simulated data using the same parameters with a rare disease or disorder with population lifetime prevalence of *K*=0.01 and a proportion of cases in the sample of *P*=0.5. The number of records (*N*) is 3000 and the true heritability is 0.5. The observed values are in excellent agreement with the expected values that are 0.93, 0.89, 0.81, 0.73 and 0.68 for the effective population size of 100, 500, 2000, 5000 and 10000..

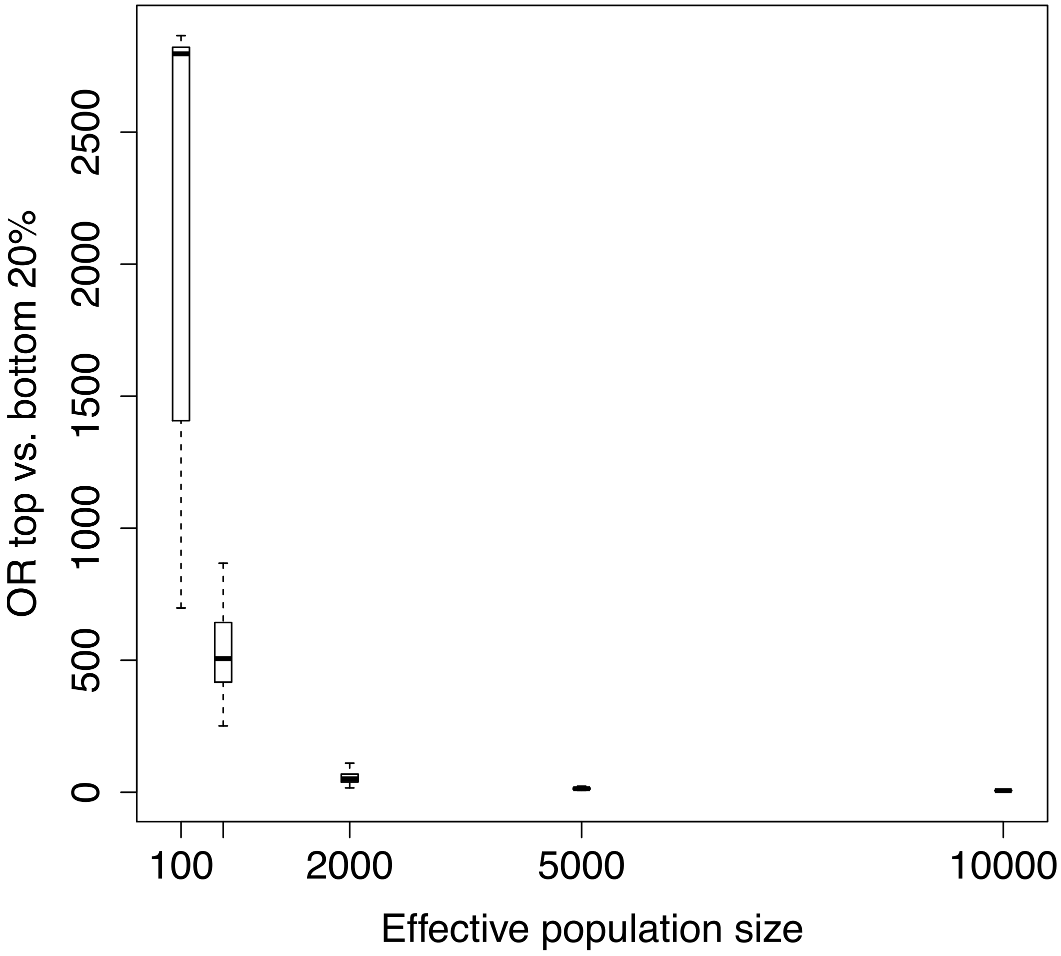


Supplementary Figure 8. Observed odd ratio contrasting the top and bottom 20% of the risk profile scores from simulated data using the same parameters with a rare disease or disorder with population lifetime prevalence of $K$=0.01 and a proportion of cases in the sample of $P$=0.5. The number of records ($N$) is 3000 and the true heritability is 0.5. The observed values coincide with the expected values that are 2000.6, 370.5, 43.3, 12.9 and 6.5 for the effective population size of 100, 500, 2000, 5000 and 10000.

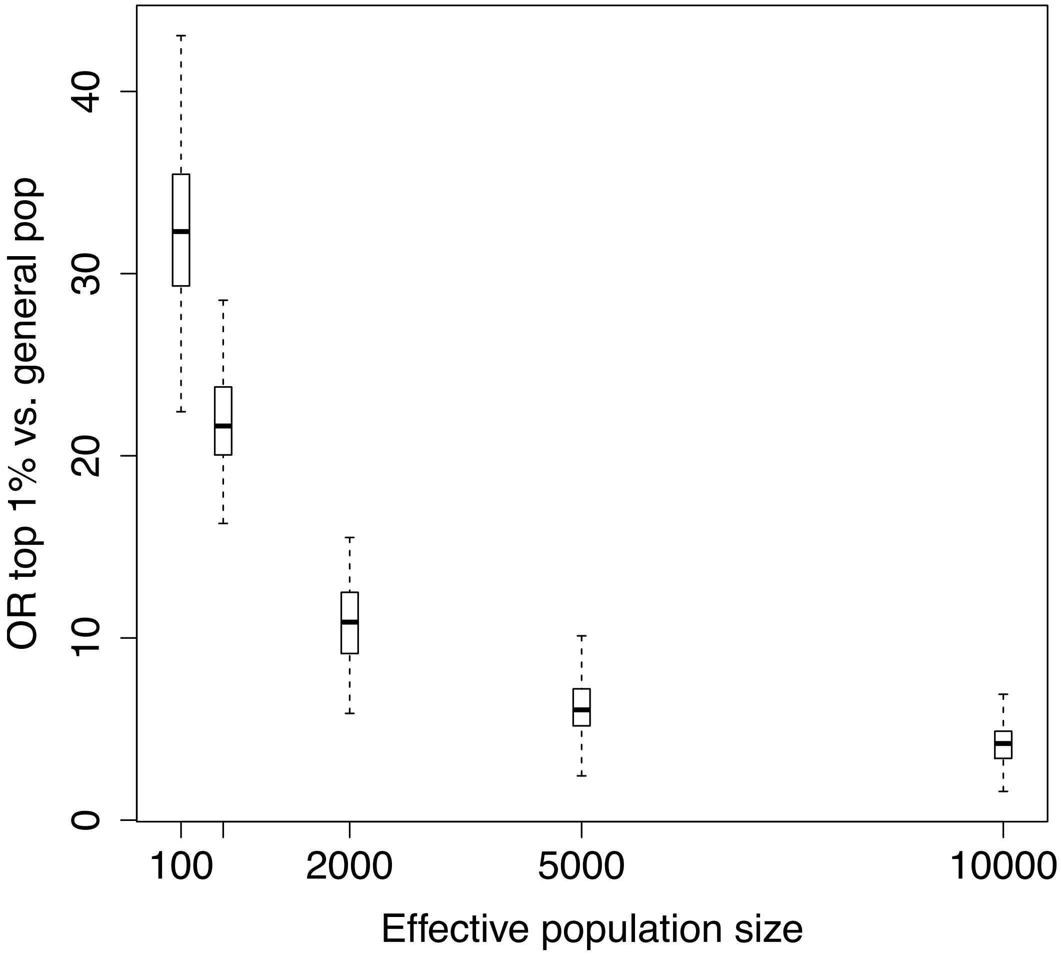


Supplementary Figure 9. Observed odd ratio contrasting the top 1% of the risk profile scores and the general population from simulated data using the same parameters with a rare disease or disorder with population lifetime prevalence of $K$=0.01 and a proportion of cases in the sample of $P$=0.5. The number of records ($N$) is 3000 and the true heritability is 0.5. The observed values are in good agreement with the expected values that are 32.5, 21.9, 10.9, 6.3 and 4.3 for the effective population size of 100, 500, 2000, 5000 and 10000.

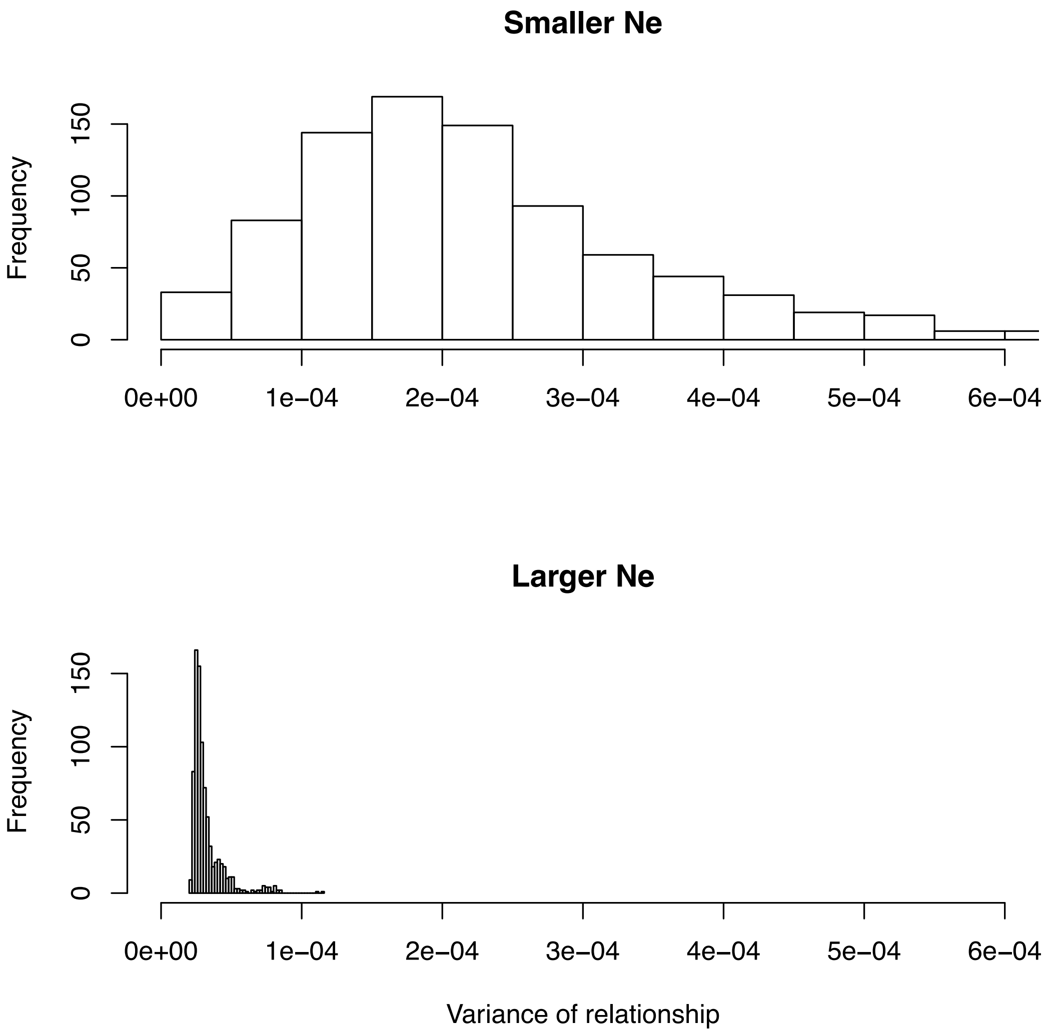


Supplementary Figure 10. The distribution of variance of relationships, paired with discovery individuals, calculated for each target individual from a design with smaller or larger $N_e$ values in a Framingham data analysis. The inferred Me is ~ 4000 and 30000 for the design with smaller and larger Ne, respectively.

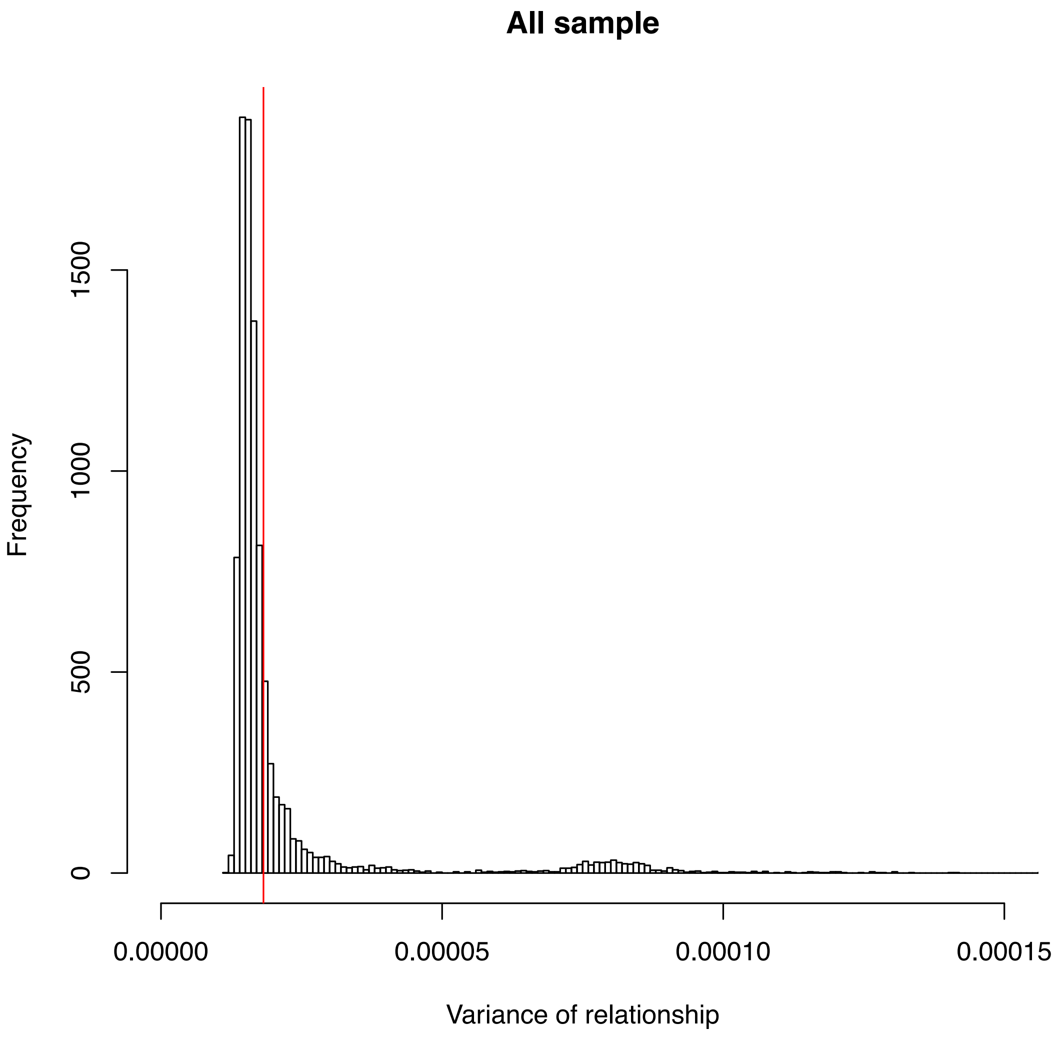


Supplementary Figure 11. The distribution of variance of relationships, paired with discovery individuals, calculated for each target individual in a GERA data analysis. The right side from the vertical line is the variance for the top 25% of the target individuals. The inferred $M_e$ decreases from ~ 58000 for the entire sample to ~ 37000 for the top 25%.

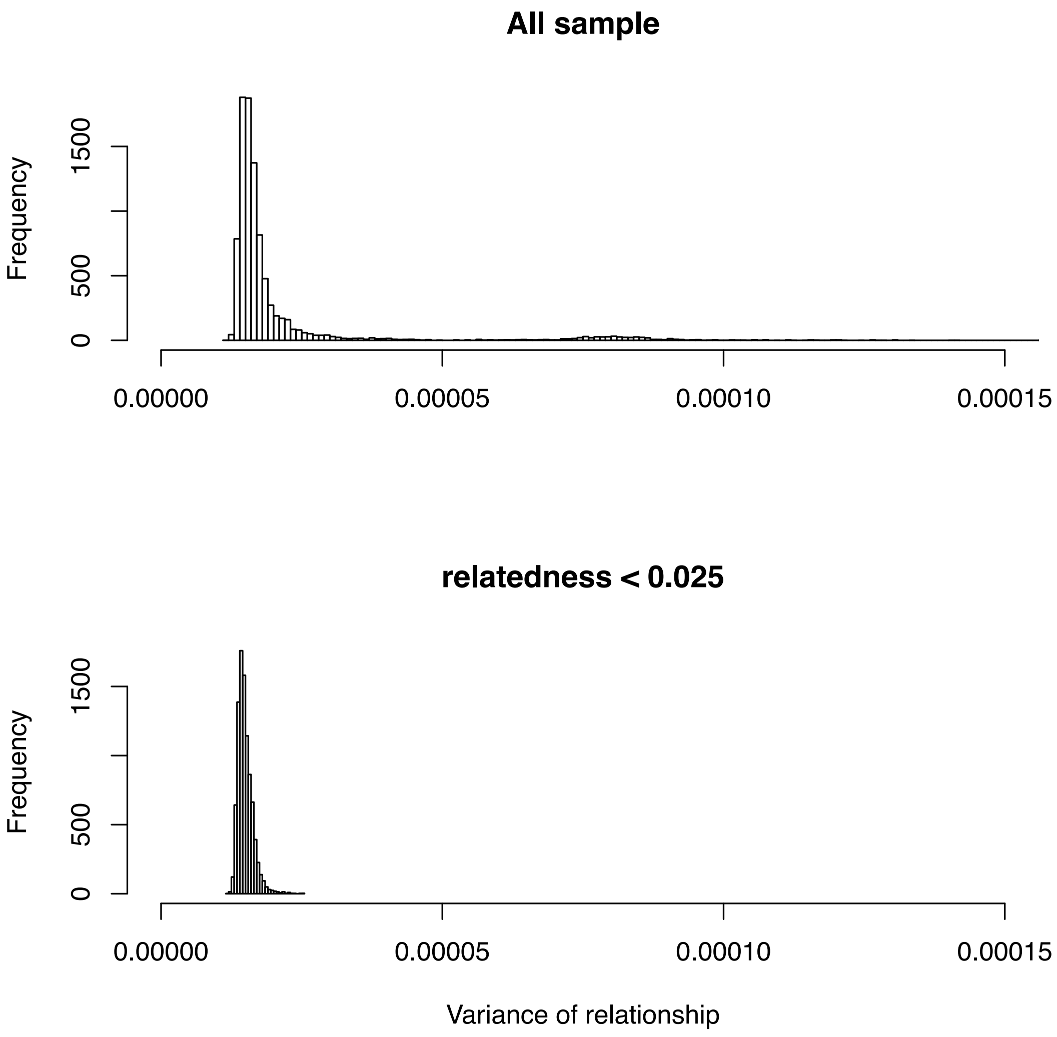


Supplementary Figure 12. The distribution of variance of relationships, paired with discovery individuals, calculated for each target individual from a design with all samples or that without relatedness > 0.025 in a GERA data analysis. The inferred $M_e$ is ~ 58000 and 67000 for the all sample and that without relatedness > 0.025, respectively.

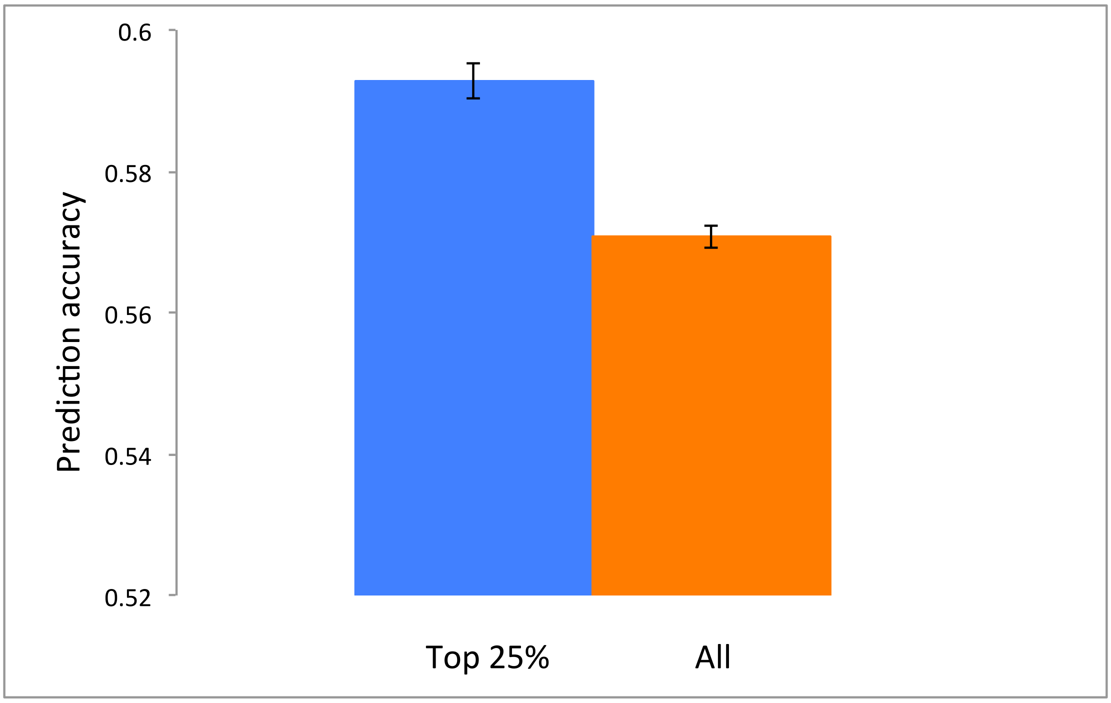


Supplementary Figure 13. The prediction accuracy is increased when using the top 25% of the target sample according to the variance of pair-wise relationships to the discovery sample. This is from a phenotypic simulation based on the real genotype data (GERA) with a heritability of 1 (the total variance fully explained by the SNPs) in order to support the result from the real data analysis (Figure 6) that the higher accuracy for the top 25% group was not due to non-genetic effects. The error bar shows the 95% confidence interval of the observed prediction accuracy over 100 replicates.

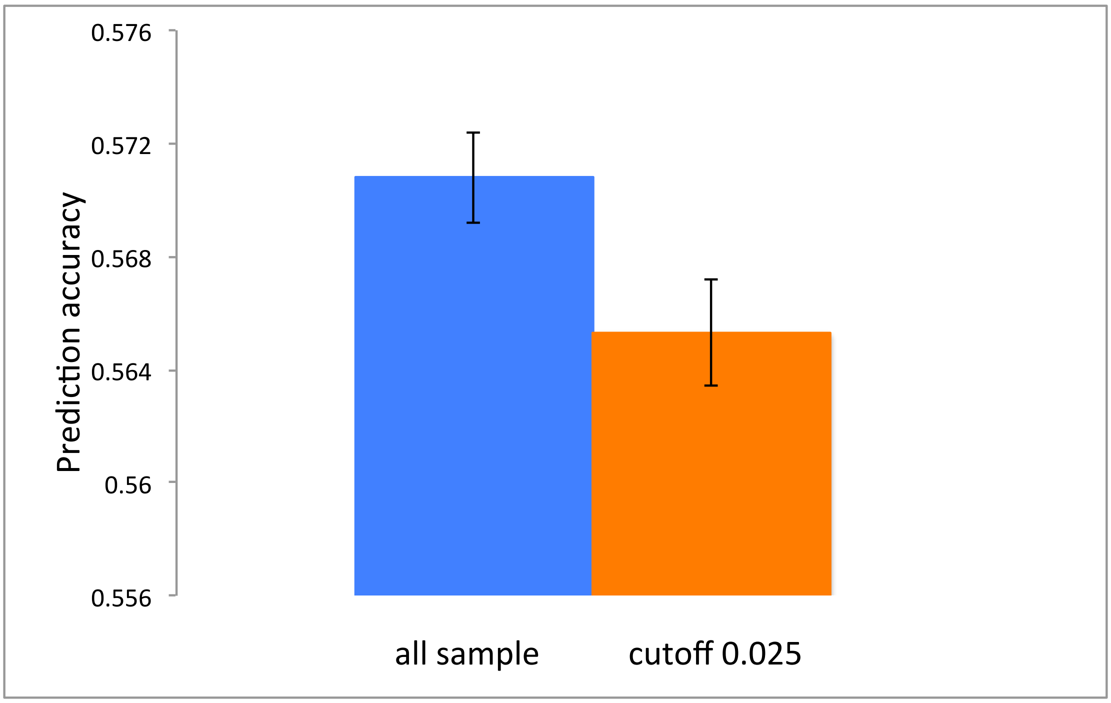


Supplementary Figure 14. The prediction accuracy is significantly decreased when excluding higher relationships from the sample that results in increasing $M_e$ (from 58000 to 67000) when using a phenotypic simulation based on the real genotype data (GERA) with a heritability of 1 (the total variance fully explained by the SNPs) in order to support the result from the real data analysis (Figure 7) in that the lower accuracy when excluding higher relatedness was not due to non-genetic effects. The same number of discovery and target sample is used for both tests. The error bar shows the 95% confidence interval of the observed prediction accuracy over 100 replicates.

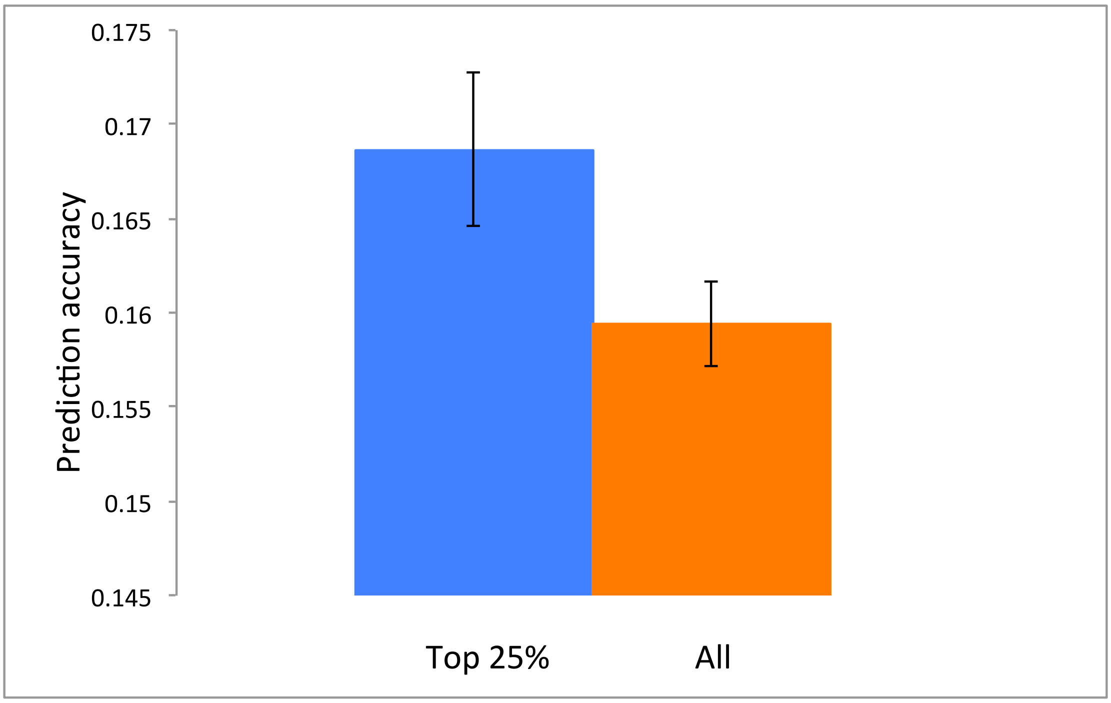


Supplementary Figure 15. The prediction accuracy is increased when using the top 25% of the target sample according to the variance of pair-wise relationships to the discovery sample. This is from a phenotypic simulation based on the real genotype data (GERA) with a heritability of 0.25 (25% of the total variance explained by the SNPs) in order to support the result from the real data analysis (Figure 6) that the higher accuracy for the top 25% group was not due to non-genetic effects. The error bar shows the 95% confidence interval of the observed prediction accuracy over 100 replicates.

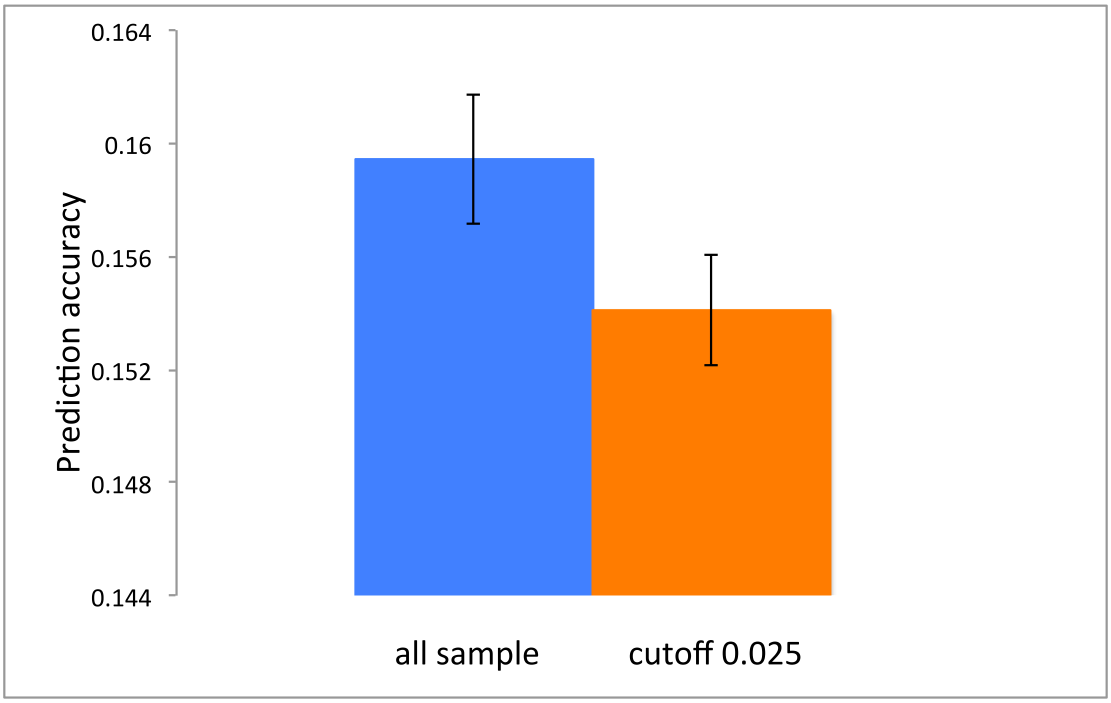


Supplementary Figure 16. The prediction accuracy is significantly decreased when excluding higher relationships from the sample that results in increasing $M_e$ (from 58000 to 67000) when using a phenotypic simulation based on the real genotype data (GERA) with a heritability of 0.25 (25% of the total variance explained by the SNPs) in order to support the result from the real data analysis (Figure 7) in that the lower accuracy when excluding higher relatedness was not due to non-genetic effects. The same number of discovery and target sample is used for both tests. The error bar shows the 95% confidence interval of the observed prediction accuracy over 100 replicates.

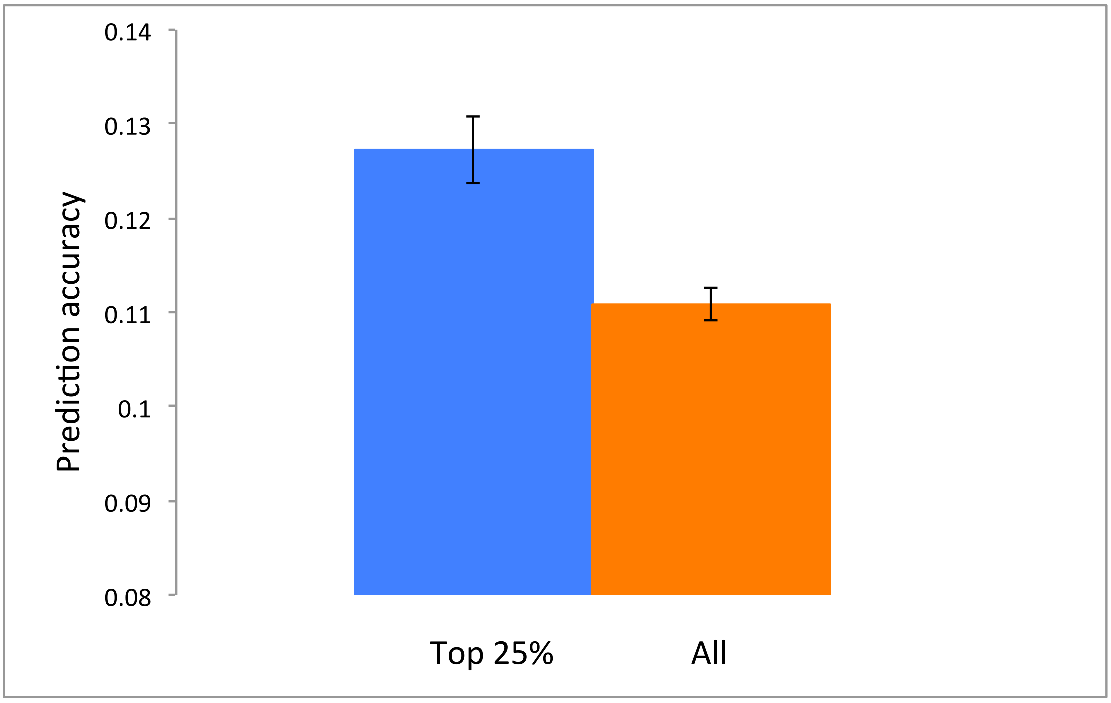


Supplementary Figure 17. The prediction accuracy is significantly increased when using the top 25% of the target sample according to the variance of pair-wise relationships with the discovery sample (therefore decreasing $M_e$ from 58000 to 37000). GERA data with dyslipidemia phenotypes are used. The error bar shows the 95% confidence interval of the observed prediction accuracy over 100 replicates.

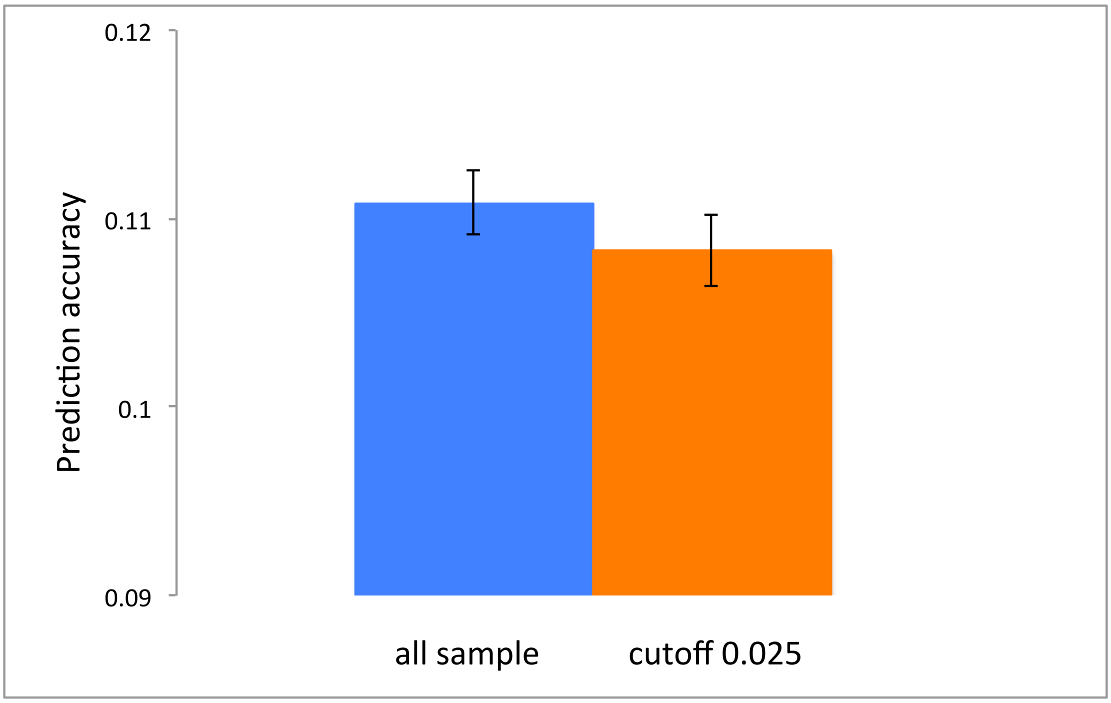


Supplementary Figure 18. The prediction accuracy is decreased when excluding higher relationships from the sample that results in increasing $M_e$ (from 58000 to 67000). GERA data with dyslipdemia phenotypes are used. The same number of discovery and target sample is used for both tests. The error bar shows the 95% confidence interval of the observed prediction accuracy over 100 replicates.

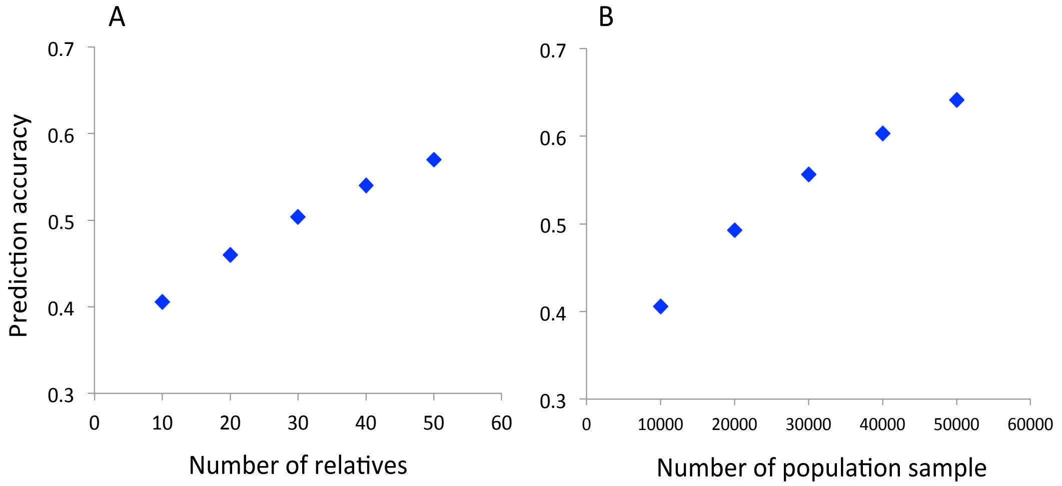


Supplementary Figure 19. The prediction accuracy is increased when additional information is used. It is assumed that the heritability of the trait is 0.5. **A**. Given that the discovery data have 10,000 individuals that are distantly related to the target sample, adding relatives (relationship of 0.125) increases the prediction accuracy. **B**. Given that the discovery data have 10 relatives (relationship of 0.125), adding more distantly related individuals (half of them have relationship of 0 and the other half have relationship of 0.01 with the target sample) improves the prediction accuracy.

References


1. de los Campos, G., Vazquez, A.I., Fernando, R., Klimentidis, Y.C., and Sorensen, D. (2013). Prediction of Complex Human Traits Using the Genomic Best Linear Unbiased Predictor. PLoS Genet 9, e1003608.
2. Lee, S.H., and van der Werf, J.H.J. (2016). MTG2: An efficient algorithm for multivariate linear mixed model analysis based on genomic information. Bioinformatics 10.1093/bioinformatics/btw012.
3. Falconer, D.S., and Mackay, T.F.C. (1996). Introduction to quantitative genetics.(Harlow, Essex, UK: Longman).
4. Kotz, S., Johnson, N.L., and Balakrishnan, N. (2000). Continuous multivariate distributions: Volume 1.(New York: Wiley).
5. Goddard, M.E., Hayes, B.J., and Meuwissen, T.H.E. (2011). Using the genomic relationship matrix to predict the accuracy of genomic selection. Journal of Animal Breeding and Genetics 128, 409-421.
6. Sved, J.A. (1971). Linkage disequilibrium and homozygosity of chromosome segments in finite populations. Theor Popul Biol 2, 125-141.
7. Tenesa, A., Navarro, P., Hayes, B.J., Duffy, D.L., Clarke, G.M., Goddard, M.E., and Visscher, P.M. (2007). Recent human effective population size estimated from linkage disequilibrium. Genome Research 17, 520-526.
8. Goddard, M.E. (2009). Genomic selection: prediction of accuracy and maximisation of long term response. Genetica 136, 245-257.
9. Meuwissen, T., Hayes, B., and Goddard, M. (2013). Accelerating Improvement of Livestock with Genomic Selection. Annual Review of Animal Biosciences 1, 221-237.
10. Elks, C.E., den Hoed, M., Zhao, J.H., Sharp, S.J., Wareham, N.J., Loos, R.J., and Ong, K.K. (2012). Variability in the heritability of body mass index: a systematic review and meta-regression. Frontiers in endocrinology 3, 29.
11. Wilson, J.G., Rotimi, C.N., Ekunwe, L., Royal, C.D., Crump, M.E., Wyatt, S.B., Steffes, M.W., Adeyemo, A., Zhou, J., Taylor, H.A., Jr., et al. (2005). Study design for genetic analysis in the Jackson Heart Study. Ethnicity & disease 15, S6-30-37.
12. Vattikuti, S., Guo, J., and Chow, C.C. (2012). Heritability and genetic correlations explained by common SNPs for metabolic syndrome traits. PLoS Genet 8, e1002637.
13. Zaitlen, N., Pasaniuc, B., Sankararaman, S., Bhatia, G., Zhang, J., Gusev, A., Young, T., Tandon, A., Pollack, S., Vilhjalmsson, B.J., et al. (2014). Leveraging population admixture to characterize the heritability of complex traits. Nat Genet 46, 1356-1362.
